# Molecular Insights into the Potential Cardiometabolic Effects of GLP-1 Receptor Analogs and DPP-4 Inhibitors

**DOI:** 10.3390/ijms26146777

**Published:** 2025-07-15

**Authors:** Małgorzata Król, Patrycja Kupnicka, Justyna Żychowska, Patrycja Kapczuk, Izabela Szućko-Kociuba, Eryk Prajwos, Dariusz Chlubek

**Affiliations:** 1 Department of Biochemistry and Medical Chemistry, Pomeranian Medical University in Szczecin, Powstańców Wlkp. 72, 70-111 Szczecin, Poland; 2 Institute of Biology, University of Szczecin, 13 Wąska, 71-415 Szczecin, Poland

**Keywords:** glucagon-like peptide-1, GLP-1 receptor agonist, cardiovascular diseases, dipeptidyl peptidase-4 inhibitor, inflammation, oxidative stress, mitochondrial function, diabetes

## Abstract

Cardiovascular diseases (CVDs) are the leading cause of global mortality, with type 2 diabetes mellitus (T2DM) and obesity significantly increasing the risk of CVD. Glucagon-like peptide-1 receptor agonists (GLP-1 RAs) and dipeptidyl peptidase-4 inhibitors (DPP-4is) have gained attention for their potential cardioprotective effects. Therefore, this review aims to explore the molecular mechanisms underlying the cardiovascular benefits of these agents. A literature review was conducted searching PubMed databases from 1990 to January 2025, including research on the effects of GLP-1 RA and DPP-4i on cardiovascular health, specifically concerning atherosclerosis, coronary artery disease, vascular health, cardiac arrhythmias, myocardial infarction (MI), and heart failure, with a focus on the biochemical and molecular effects of these drugs. We analyzed 131 scientific publications, which indicate that GLP-1 RA and DPP-4i significantly reduce cardiovascular risk and major adverse cardiovascular events (MACEs), including atherosclerosis, myocardial infarction, and cardiac arrhythmias. These clinical outcomes are attributed to the mitigation of oxidative stress, inflammation, and endothelial dysfunction as well as improvement in mitochondrial function and lipid metabolism. GLP-1 RAs offer substantial cardiovascular benefits, making them valuable in managing T2DM and reducing CVD risk. Their integration into treatment regimens for CVD can reduce hospitalization rates, improve quality of life, and extend life expectancy. DPP-4is, while beneficial, are less effective in cardiovascular protection. Further research is needed to optimize therapeutic strategies and broaden the clinical application of these agents in cardiometabolic care.

## 1. Introduction

Cardiovascular diseases (CVDs) are the leading cause of mortality, premature death, and rising healthcare costs [1,2,3]. CVDs encompass multiple conditions, including coronary artery disease (CAD), cerebrovascular disease (CD), peripheral artery disease (PAD), and aortic atherosclerosis [4], all of which can lead to heart failure, angina, and myocardial infarction. In cardiovascular research, the acronym MACE (major adverse cardiovascular events) is commonly used to describe combined endpoints that include myocardial infarction, stroke, and cardiovascular death (3P-MACE), or additionally, angina pectoris (4P-MACE) [5,6,7].

The pathophysiology of cardiovascular diseases involves multiple interconnected processes, including increased free radical production, inflammation, and mitochondrial dysfunction, affecting both cardiac tissue and the vascular system. Oxidative stress induced by excessive free radicals leads to damage to cardiac cells, which can result in heart failure. Additionally, free radicals can activate signaling pathways that promote apoptosis and fibrosis within the cardiac muscle [8]. Oxidative stress also impairs mitochondrial function in cardiomyocytes, which is essential for the adenosine triphosphate (ATP) production necessary for cardiac contraction. A reduction in ATP production consequently weakens cardiac function [8,9]. Mitochondria also play a key role in calcium homeostasis. In the heart muscle, Ca^2+^ is crucial for generating electrical signals. Excess free radicals disrupt the electrolyte balance in cardiac cells, potentially altering ion channels and impairing the flux of calcium, sodium, and potassium ions, leading to abnormal contractions and arrhythmias [10].

Various oxidative enzyme systems, including nicotinamide adenine dinucleotide phosphate (NADPH) oxidase, xanthine oxidase, cyclooxygenases, lipoxygenases, myeloperoxidases, cytochrome P450 monooxygenase, uncoupled nitric oxide synthase (NOS), and peroxidases, contribute to nitric oxide (NO) inactivation. This process might be a key driver of the endothelial dysfunction [11] observed in cardiovascular diseases. Oxidized low-density lipoprotein (ox-LDL) promotes atherosclerotic plaque formation by activating endothelial cells, inducing macrophage-driven foam cell formation, and stimulating the migration and proliferation of smooth muscle cells (SMCs) [12], all of which can contribute to myocardial infarction. Ischemia further exacerbates mitochondrial damage, reducing ATP production while increasing the generation of reactive oxygen species (ROS). Upon reperfusion, damaged mitochondria can exacerbate cellular injury by producing excessive ROS.

A persistent global increase in the incidence of CVD has been observed for many years. In Europe, cardiovascular diseases account for the loss of more than 60 million years of life [13]. Primary prevention strategies aim to avert the occurrence of cardiovascular events in individuals identified as having significant risk factors but no prior history of such conditions. Secondary prevention focuses on implementing therapeutic interventions to mitigate further cardiac damage and reduce the likelihood of recurrent events in patients with a documented history of CAD [14].

The implementation of novel, comprehensive therapies for the prevention and treatment of CVD is imperative. A deeper understanding of the molecular mechanisms underlying the actions of new pharmacological agents will enable their safe integration into treatment strategies for patients with multiple comorbidities.

### Mechanisms of Action and Clinical Indications of GLP-1 Receptor Agonists (GLP-1 RAs) and DPP-4 Inhibitors (DPP-4is)

Effective glycemic control and appropriate type 2 diabetes mellitus (T2DM) treatment lower the risk of CVD. Among the most cardioprotective antidiabetic drugs, sodium–glucose cotransporter 2 inhibitors (SGLT2is) are commonly prescribed for patients with elevated CVD risk. Recently, glucagon-like peptide-1 receptor agonists (GLP-1 RAs) and dipeptidyl peptidase-4 inhibitors (DPP-4is) have garnered significant attention [15].

GLP-1 RAs mimic the action of natural incretin hormones, which are secreted from K cells (gastric inhibitory polypeptide, GIP) and L cells (glucagon-like peptide-1) of the intestines in response to food intake. Glucagon-like peptide-1 acts as a physiological modulator of insulin gene expression, enhances insulin secretion, and increases intracellular cyclic adenosine monophosphate (cAMP) levels, influencing the activity of multiple proteins and transcription factors [16]. GLP-1 and its analogs reduce gastric acid secretion, slow gastric emptying, and inhibit glucagon release. T2DM patients exhibit lower endogenous GLP-1 levels, which may contribute to poor glycemic control and increased CVD risk [17,18].

GLP-1 RAs are currently used not only for diabetes management but also as pharmacotherapy for overweight, obesity, and cardiovascular protection [19,20]. These drugs function by binding to the GLP-1 receptor (GLP-1R), a G protein-coupled receptor. GLP-1R is highly expressed in various cardiac tissues and the vascular system. Its messenger ribonucleic acid (mRNA) expression has been detected in murine endocardial cells [21], atrial cardiomyocytes [22], and porcine pacemaker cells of the sinus node [23]. Ban et al. (2008) demonstrated GLP-1R mRNA expression in cardiomyocytes, endothelial cells, and SMCs, along with translated GLP-1R protein in mesenteric artery SMCs and an aortic SMC line [24]. In human tissues, GLP-1R mRNA expression has been observed in atrial and ventricular cardiomyocytes [21] as well as in the sinoatrial node [25]. Low GLP-1R mRNA expression has also been detected in nerves, fibroblasts, and immune cells of the ventricles [26]. In the vascular system, GLP-1R is found in SMCs of ventricular blood vessels and tibial and thoracic arteries in mice [21] as well as in the cerebrovascular system, including cortical arterioles, capillaries, and venules [27]. In human tissues, GLP-1R has been identified in vascular SMCs of renal arterioles [15] and is suspected to be present in endothelial cells of the umbilical vein and aorta [28], though findings remain inconclusive [26,29].

GLP-1 action is rapidly terminated by degradation via endogenous dipeptidyl peptidase-4 (DPP-4) [30,31]. DPP-4, classified within the CD26 cluster, is a widely expressed enzyme whose main role is to cleave dipeptides from the N-terminal end of certain proteins, including incretin hormones such as GLP-1 and GIP [32]. It also plays a critical role in glucose metabolism by enhancing insulin secretion, increasing β cell mass, and reducing glucagon secretion [33,34]. Moreover, it influences insulin sensitivity and acts as a mediator of inflammation in adipose and hepatic tissues, potentially linking obesity to the pathogenesis of type 2 diabetes [33].

DPP-4 inhibitors are a class of oral antidiabetic drugs developed for the treatment of type 2 diabetes [35]. Five DPP-4 inhibitors are available in Europe: sitagliptin, vildagliptin, saxagliptin, linagliptin, and alogliptin [36]. These agents work by inhibiting the enzyme DPP-4, which improves glycemic control by reducing both fasting and postprandial glucose levels without causing weight gain or increasing the risk of hypoglycemia [37]. Beyond their glucose-lowering properties, DPP-4is are promising agents in atherosclerosis prevention and lowering cardiovascular risk [38] (Table 1).

To sustain GLP-1’s glucose-modulating effects, GLP-1 analogs have been modified for extended activity and are classified as either short-acting (exenatide, beinaglutide, lixisenatide) [39,40] or long-acting (liraglutide, dulaglutide, albiglutide, semaglutide) [31,41]. In clinical trials, investigational drugs include Icosema [42,43], Mazdutide [44,45], Orforglipron [46], Cagrisema [46,47], Retatrutide [48], Survodutide [49], and TG103 [50]. Another available preparation is exenatide, a synthetic version of exendin-4 (Ex-4), a peptide isolated from the venom of the Gila monster, which acts as a GLP-1 RA [51,52]. Exenatide is resistant to inactivation by DPP-4 [53].

Until recently, a product of GLP-1 and its analog’s degradation, the GLP-1 (9–36), was considered inactive. However, its presence may enhance the cardioprotective effects of GLP-1 RA, in part through interactions with endothelial mitochondria in a GLP-1R-independent manner [54,55].

**Table 1:** A comparison of glucagon-like peptide-1 receptor agonists (GLP-1 RAs) and dipeptidyl peptidase-4 inhibitors (DPP-4is) in terms of their mechanistic specificity, therapeutic efficacy, clinical indications, and relevance.

	DPP-4i	GLP-1 RA
Mechanism of action	Inhibition of the DDP-4 enzyme, which breaks down GLP-1, increasing the concentration of endogenous GIP and GLP-1 [56]	Direct activation of the GLP-1 receptor; modified to be resistant to DDP-4 [57]
Effect on body weight	No significant impact [58]	Weight reduction of approximately 1–4 kg [59]
HbA1c reduction	Smaller average reduction, moderate reduction of 0.4–1.1% [60]	Greater reduction of 0.6–1.9% [59]
Risk of hypoglycemia	Low [61]	Low [61]
Route of administration	Oral [62]	Subcutaneous, oral [62]
Dosing frequency	Once daily [63]	Ranges from once daily to once weekly [63]
Common side effects	Mild: headaches, upper respiratory tract infections [63]	Usually transient diarrhea, nausea, and vomiting [63]
Clinical indications	Patients with moderate hyperglycemia, without the need for weight loss, and who prefer oral therapy will benefit.	Patients who require tighter glycemic control, are at high cardiovascular risk, and are obese will benefit.

## 2. Cardioprotective Properties of GLP-1 Analogs, Receptor Agonists, and DPP-4 Inhibitors

### 2.1. Major Adverse Cardiovascular Events

A high-quality meta-analysis involving 18 randomized controlled trials conducted by Zhang et al. demonstrated that among GLP-1 RAs, GLP-1 analogs significantly reduced MACE risk compared with Ex-4 analogs in patients with T2DM and chronic kidney disease (CKD) [64]. However, no significant differences were observed between GLP-1 analogs and SGLT2i or finerenone therapy [64]. Similarly, Lin et al., in a network meta-analysis including 21 trials with 170,930 participants, found that GLP-1 RAs were associated with a reduced risk of 3P-MACE compared with placebo or DPP-4i [65]. The benefits of GLP-1 RAs were more pronounced in elderly patients, individuals of White or Asian descent, those diagnosed with atherosclerotic cardiovascular disease (ASCVD), and patients with poor glycemic control. Notably, in another valuable meta-analysis involving over 17,000 patients, the use of vildagliptin was associated with a reduced risk of MACE in T2DM patients younger than 65 years but not in those older than 65 [66,67].

In contrast, a meta-analysis of 111,565 participants by Sohn et al. indicated that SGLT2is were more effective at reducing 3P-MACE risk among T2DM patients with reduced estimated glomerular filtration rate (eGFR) and albuminuria. However, in patients with normal eGFR, GLP-1 RA showed greater efficacy than SGLT2i [68].

An Italian cohort study, however, favored GLP-1 RA over SGLT2i. The study showed a significant reduction in 3P-MACE and 4P-MACE risk among patients using GLP-1 RAs. Although observational, these findings suggest that GLP-1 RAs are not only safe but also more effective than SGLT2is [69].

Based on high-value meta-analysis, both GLP-1 RA and SGLT2i exhibit the highest effectiveness in reducing MACE risk, significantly outperforming DPP-4i. The American Diabetes Association (ADA) ecommends the use of either SGLT2 inhibitors or GLP-1 RA in individuals with type 2 diabetes and established ASCVD or multiple ASCVD risk factors [70]. However, due to their robust nephroprotective effects, SGLT2 inhibitors are considered the preferred agents in patients with coexisting CKD, whereas GLP-1 RA may be particularly beneficial in individuals with concomitant obesity [70]. However, treatment choices should be personalized based on patient profiles and expected outcomes [65].

### 2.2. Cardiovascular Risk

Several randomized controlled trials and real-world observational studies have shown the positive effects of GLP-1 RA on cardiovascular risk factors in patients with T2DM. These effects include reductions in body weight, blood pressure, lipid levels, and inflammation markers.

In a meta-analysis of randomized controlled trials, Kan et al. demonstrated that dulaglutide, exenatide, and semaglutide significantly improved cardiovascular risk profiles in patients with T2DM complicated by coronary artery disease. These GLP-1 RAs and DPP-4is significantly reduced cardiovascular risk factors, including blood glucose levels, hemoglobin A1c (HbA1c), fasting blood glucose (FBG), body weight, body mass index (BMI), systolic blood pressure (SBP), diastolic blood pressure (DBP), total cholesterol (TC), and low-density lipoprotein cholesterol (LDL-C), compared with placebo or conventional treatment [71] Additionally, dulaglutide, when included in standard antihyperglycemic therapy for patients without documented cardiovascular disease, lowered the risk of cardiovascular events [72]. These findings suggest a potential role for GLP-1 RA and DPP-4i in both primary and secondary prevention, although the evidence supporting their use in primary prevention is currently less robust.

A retrospective cohort analysis by Riley et al. [73] found that GLP-1 RA and/or SGLT2i significantly reduced the risk of cardiovascular diseases such as acute myocardial infarction, heart failure, hospitalization risk, and all-cause mortality in T2DM patients compared with those receiving insulin alone. Although these results are based on observational data, they provide real-world evidence supporting the cardiovascular benefits of these agents.

Moreover, research has shown that GLP-1 RAs provide cardiovascular benefits even in patients without diabetes [74]. Meta-analyses indicate that GLP-1 RAs lower both systolic and diastolic blood pressure and improve lipid profiles in obese patients without T2DM, thereby reducing cardiovascular risk [74,75].

GLP-1 RAs have demonstrated the most consistent effects on weight, blood pressure, and inflammatory markers across multiple studies. While improvements in these surrogate endpoints are encouraging, their translation into a reduction in cardiovascular events depends on the patient population and baseline risk.

### 2.3. Atherosclerosis and Atherothrombosis

Endothelial dysfunction and the development of lipid-rich plaques in the walls of medium- to large-sized arteries are hallmarks of atherosclerosis, a chronic inflammatory disease. The ultimate consequence of this disease is atherothrombosis, where occlusive thrombi are formed as a result of atherosclerotic plaque erosion or rupture [76]. Atherothrombosis is the primary cause of acute coronary syndromes [77]. The development of atherosclerosis is one of the elements that contribute to coronary artery disease [78].

The impact of GLP-1 RA on deep vein thrombosis, unstable angina, and other thrombotic events remains inconclusive [74,75]. According to a meta-analysis of randomized controlled trials, the drugs appear to modulate mechanisms involved in anti-atherosclerotic effects; however, researchers showed no statistically significant reduction in deep vein thrombosis incidence.

Research by Kahal et al., involving 19 obese women with PCOS and 17 controls treated with liraglutide, demonstrated reduced inflammation, improved insulin sensitivity, and enhanced lipid homeostasis. Furthermore, liraglutide significantly reduced atherothrombosis markers in young obese women with and without polycystic ovary syndrome (PCOS). Additionally, liraglutide contributed to improved endothelial function and reduced clot lysis area, thereby playing a role in lowering cardiovascular risk [79].

#### 2.3.1. Molecular Mechanisms Involved in GLP-1 RA and DPP-4i Effects in Atherosclerosis and Atherothrombosis

##### Atherosclerotic Plaque Markers

GLP-1 RA and DPP-4i reduce the risk of atherosclerotic plaque formation by mitigating inflammation, limiting leukocyte recruitment, suppressing leukocyte rolling and adhesion, and modulating extracellular matrix protein turnover [80,81,82]. These findings are predominantly based on preclinical studies.

A controlled clinical trial by Kahal et al. demonstrated that liraglutide significantly reduced atherothrombosis markers, including P-selectin, soluble intercellular adhesion molecule (sICAM), and soluble vascular cell adhesion molecule (sVCAM), in young obese women with and without PCOS [79]. However, the sample size was small and limited to a non-cardiovascular population [79].

Atherosclerotic plaque might contain LPS, whose proinflammatory action contributes to plaque instability and the formation of microthrombi. Endotoxemia may be associated with intestinal dysbiosis and changes in intestinal permeability, which often coexist with people suffering from obesity, a population at high cardiovascular risk [83,84]. Lipopolysaccharide (LPS) activates innate immune responses by stimulating neutrophils and macrophages to produce proinflammatory cytokines and mediators [85,86]. This response is mediated via the toll-like receptor 4 (TLR4) and myeloid differentiation factor (MD2) complex. Upon binding of LPS to the TLR4/MD2 complex, several adaptor molecules are recruited, including primary myeloid differentiation response 88 (MyD88). MyD88 activates the nuclear factor kappa–light-chain-enhancer of activated B cells (NF-κB). As a consequence of this action, proinflammatory genes are transactivated, including IL-12, cyclooxygenase-2 (COX-2), tumor necrosis factor alpha (TNF-α), and inducible nitric oxide synthase (iNOS) [83,84,87].

In in vivo studies using LPS-induced endotoxemia, both gliptins and liraglutide decreased the mRNA expression of the adhesion molecules vascular cellular adhesion molecule-1 (VCAM-1) and intercellular adhesion molecule-1 (ICAM-1) and downregulated inflammatory mediators, including interleukin 6 (IL-6), monocyte chemoattractant protein-1 (MCP-1), and TNF-α in aortic tissue. These protective and anti-inflammatory effects are likely mediated by adenosine monophosphate (AMP)-activated protein kinase (AMPK) activation in DPP-4i treatment [88]. Similar findings were observed in endothelial cells, where liraglutide treatment mitigated ox-LDL-induced cytotoxicity and suppressed ICAM-1 and VCAM-1 expression [89].

Additionally, DPP-4i reduced foam cell formation of macrophages isolated from T1D mice and patients [90] and mitigated inflammation [38] and plaque instability in in vivo studies [91].

##### Extracellular Matrix Remodeling

In atherosclerosis, extracellular matrix (ECM) remodeling is regulated by matrix metalloproteinases (MMPs) and tissue inhibitors of metalloproteinases (TIMPs) [92]. In an in vitro study, GLP-1 RA not only downregulated VCAM-1 and ICAM-1 but also restored TIMP-1 and TIMP-2 balance while regulating MMP-1 and MMP-2 levels in coronary artery endothelial cells [93]. These effects influence atherosclerotic plaque vulnerability and contribute to improved endothelial function [93].

##### Oxidative Stress and Endothelial Function

The oxidation of low-density lipoprotein (ox-LDL) plays a pivotal role in atherosclerosis initiation and progression [94]. In a preclinical in vivo model using fructose-fed rats, Wójcicka et al. demonstrated that a four-week exenatide treatment significantly increased plasma platelet-activating factor acetylhydrolase (PAF-AH) activity while reducing circulating ox-LDL and MCP-1 levels. These changes were accompanied by a decrease in the phosphatidylcholine (PC)/lysophosphatidylcholine (lyso-PC) ratio and an improved apolipoprotein B (apoB)/apolipoprotein A-I (apoA-I) balance. Notably, exenatide—but not sitagliptin—prevented LDL oxidation, likely due to its favorable impact on PAF-AH activity [95]. Similarly, Ying et al., using an in vivo model of atherosclerosis, found that liraglutide effectively reduced lectin-like oxidized low-density lipoprotein receptor 1 (LOX-1) expression in aortic endothelial cells and decreased oxidative stress and inflammation in LDL receptor-deficient (LDLR-KO) mice. These effects were reversed by exendin-9, indicating a direct role of GLP-1R activation [89]. In ox-LDL-induced endothelial dysfunction, liraglutide also reduced ROS levels, apoptosis, and the upregulation of LOX-1, nicotinamide adenine dinucleotide phosphate oxidase 4 (NOX4), and NF-κB, suggesting that GLP-1 RAs slow atherosclerosis progression. These effects were absent when GLP-1R was silenced or LOX-1 was overexpressed, reinforcing that liraglutide enhances endothelial function through GLP-1R-mediated LOX-1 inhibition, thereby reducing oxidative stress and inflammation [89].

Accelerated atherothrombosis in patients with T2D may be associated with platelet reactivity dysfunction. Platelets from patients with T2D show increased levels of oxidative stress through the activation of oxidase 2 (NOX2) and NADPH oxidase, leading to increased ROS production [96]. GLP-1 metabolites and analogs have been shown to reduce oxidative stress by decreasing ROS production by platelets [97,98]. Additionally, GLP1-RA therapy reduced NOX2 levels and contributed to reduced sP-selectin levels, which contributed to a decrease in platelet activation [99,100].

The cardioprotective properties of GLP-1 RA and/or DPP-4i result from the reduction in oxidative stress by lowering ROS levels [101,102], increasing superoxide dismutase activity and glutathione peroxidase (GPx) activity [103], and downregulating inducible nitric oxide synthase (iNOS), as demonstrated in animal models and in vitro studies. Additionally, sitagliptin and liraglutide influenced the endothelial nitric oxide synthase (eNOS) coupling state, leading to a mixed coupling effect in endotoxemic animals after treatment [88]. In in vitro experiments using human macrophages, exenatide treatment reduced both ROS and malondialdehyde (MDA) levels, a marker of lipid peroxidation and oxidative damage [103]. Furthermore, lixisenatide promoted the expression of antioxidant regulators such as nuclear factor erythroid 2-related factor 2 (Nrf2) and heme oxygenase-1 (HO-1) in primary human umbilical vein endothelial cells (HUVECs) [104]. In the hyperhomocysteinemia animal model, a risk factor for atherosclerosis, exenatide attenuated endothelial dysfunction by reducing oxidative stress through the AMPK/eNOS pathway [105].

Moreover, liraglutide treatment enhanced eNOS expression [106] and decreased its uncoupling in vivo [80]. eNOS plays a cardioprotective role, and its uncoupling contributes to decreased levels of tetrahydrobipterin (BH4) and endothelial dysfunction in aging vessels [107]. Interestingly, sitagliptin demonstrated inferior results compared with linagliptin in studies assessing NO production and vascular function [88]. In cardiac microvascular endothelial cells (CMECs), GLP-1 RA reduced ROS production, apoptosis rates, and NADPH oxidase components (p47(phox) and gp91(phox)) under high-glucose conditions in vitro [108]. Additionally, the vascular oxidative stress reduction induced by liraglutide has been linked to increased Nrf2 nuclear translocation and AMPK phosphorylation [109].

Steven et al. demonstrated that DPP-4i and GLP-1 RA improved vascular function in isolated rat aortas through enhanced cyclic guanosine monophosphate (cGMP)-dependent kinase activity and NO/cGMP signaling [88]. This results in vascular relaxation and contributes to the improvement of endothelial function and vasodilation [110]. However, NO levels also increased in the LPS group without corresponding cGMP activity enhancement, suggesting that the vasodilatory effects of gliptins are independent of muscarinic receptor activation [88]. In ox-LDL-treated human aortic endothelial cells (HAECs), dulaglutide restored Krüppel-like factor 2 (KLF2) transcription factor expression and prevented p53 phosphorylation in vitro [111]. KLF2 regulates eNOS expression in endothelial cells, promoting NO release and vasodilation. Furthermore, KLF2-driven genes contribute to anti-thrombotic, antioxidant, and anti-inflammatory mechanisms, effectively inhibiting atherosclerosis development and progression [112].

Additionally, sitagliptin and exenatide improved endothelial function in the aortas of diabetic rats. This effect is associated with the receptor of advanced glycation end product (RAGE) suppression and subsequent downregulation of the Rho/ROCK-induced NF-κB/NF-κB inhibitor alpha (IκBα) signaling pathway. The treatments reversed high-fat diet-induced increases in RAGE, Rho-associated coiled-coil-containing protein kinase 2 (ROCK-2), endothelin-1 (ET-1), and iNOS in vivo, as well as oxidative stress and subsequent NADPH oxidase activation, while restoring GLP-1R, phosphorylated endothelial nitric oxide synthase (p-eNOS), and phosphorylated AMPK (p-AMPK) expression [113]. Increased expression of proinflammatory mediators induced by AGE/RAGE is associated with endothelial dysfunction [114]. The Rho/ROCK signaling pathway plays a critical role in endothelium-dependent relaxation, and inhibition of Rho kinase significantly reduces vascular damage in diabetes. AGE/RAGE activation stimulates the RhoA/ROCK signaling-induced NF-κB system in the aortas of diabetic rats, leading to impaired endothelium-dependent relaxation. RhoA/ROCK activation may enhance NF-κB pathway activity. Therefore, the endothelial protective effects of GLP-1 treatment may be attributed to its RAGE-suppressing properties [113,114]. Also, DPP-4is enhance endothelial senescence by activating the AMPK/SIRT1/Nrf2 signaling pathway in in vitro studies [115].

##### Apoptosis and Inflammation

GLP-1 RA also reduced apoptosis and downregulated inflammatory markers such as IL-6, interleukin-1 beta (IL-1β), and TNF-α [102,111,116,117,118,119,120] as well as phosphorylated NF-κB and phosphorylated IκBα (p-IκBα) in various preclinical models [113]. These agents suppressed the expression of toll-like receptor 2 (TLR2), TLR4, c-Jun N-terminal kinase 1 (JNK-1), and suppressor of cytokine signaling 3 (SOCS-3), reducing proinflammatory signaling cascades in endothelial and vascular smooth muscle cells [93,119]. Also, it inhibited TNF-α-induced apoptosis, contributing to endothelial protection [93,119]. Additionally, semaglutide treatment reduced the expression of genes of chemokine (C-X-C motif) ligand 2 (Cxcl2), S100 calcium binding protein A8 (S100a8), and S100 calcium binding protein A9 (S100a9) [102] in murine models of obesity and vascular inflammation.

Liraglutide improved endothelial function and suppressed atherogenesis, and this effect was mediated by GLP-1 receptor activation [106,121]. Liraglutide was shown to reduce apoptosis in vitro and protect against high-glucose-induced vascular SMC migration and proliferation. Additionally, it promoted the phosphorylation of protein kinase B (Akt) and extracellular signal-regulated kinase 1/2 (ERK1/2) [121], key regulators of cellular survival and proliferation, regulating cardiac structure and function [122]. In apolipoprotein E (apoE)−/− mice, treatment with Ex-4 decreased monocyte adhesion to the endothelium. Moreover, it lowered TNF-α and MCP-1 levels and inhibited nuclear translocation of p65, a component of NF-κB, through the modulation of cAMP and protein kinase A (PKA) activity in endothelial cells [123]. Interestingly, in liraglutide-treated C11-STH cells, this effect appears to be mediated by a PKA-independent signaling pathway [106].

Ex-4 treatment also contributed to reduced neointimal hyperplasia following arterial injury in vivo and inhibited SMC proliferation via the cAMP/PKA pathway in vitro. It also attenuated TNF-α production by isolated peritoneal macrophages [124]. GLP-1 RA administration increased cAMP/PKA activity while decreasing Rho expression in CMEC, suggesting that the protective effects of GLP-1 are mediated through the downstream inhibition of Rho via the cAMP/PKA pathway in in vitro models [108].

##### Mitochondrial Function

GLP-1 RAs exert cardioprotective effects partly through the enhancement of mitochondrial function, including mitochondrial respiration, in cross-sectional observational studies involving patients with T2DM [80,81]. Luna-Marco et al. demonstrated that GLP-1 RA reduced reactive oxygen species production, restored mitochondrial membrane potential, and increased oxygen consumption in peripheral blood polymorphonuclear leukocytes (PMNs) isolated from T2DM patients in an ex vivo human model [80,81]. In ox-LDL-treated HAECs, dulaglutide also improved mitochondrial membrane potential. Given that mitochondrial dysfunction contributes to oxidative stress and endothelial damage, enhancing mitochondrial membrane potential may help attenuate atherogenesis in individuals with elevated ox-LDL levels [125].

GLP-1 RAs reduce apoptosis and downregulate the levels of key inflammatory factors while suppressing the expression of key upstream immune receptors responsible for the initiation of the inflammatory response. Additionally, GLP-1 RA treatment significantly attenuates oxidative stress-induced endothelial dysfunction by diminishing ROS production and increasing the expression and activation of key antioxidant enzymes. Moreover, they enhance intracellular signaling via phosphorylation of Akt and ERK1/2, increase cAMP/PKA activity, and improve mitochondrial function, contributing to their cardioprotective effects. These diverse signaling mechanisms highlight the pleiotropic antioxidant, anti-inflammatory, and endothelial-protective effects of GLP-1 RAs. However, most current data come from preclinical studies, both in vitro and in vivo. More high-quality clinical research is needed to confirm the relevance of these mechanisms.

The molecular and biochemical effects of GLP-1 RAs and DPP-4is have been summarized in Table 2.

**Figure 1:**
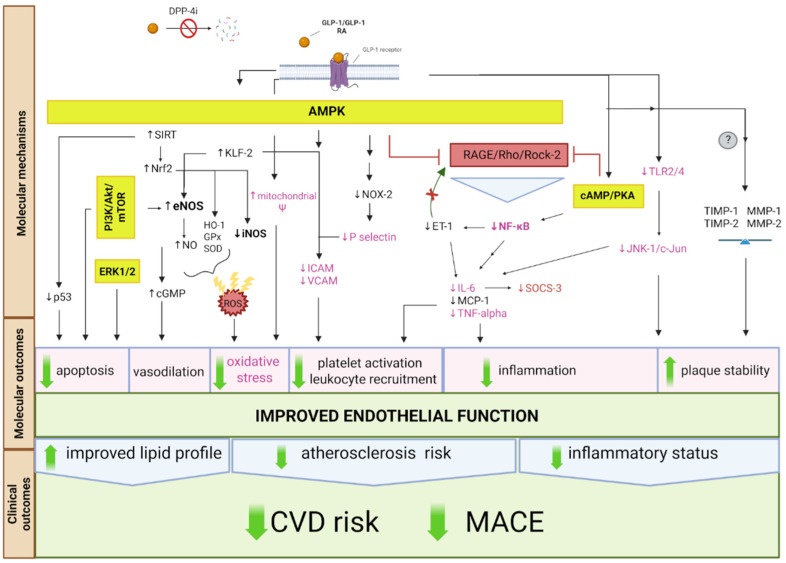
The key molecular mechanisms along with the associated molecular and clinical outcomes of GLP-1 RA and DPP-4i treatment that contribute to reduced atherosclerosis and CVD risk. After GLP-1R engagement, the activity of PI3K/Akt/mTOR and ERK1/2 signaling pathways increases, which, along with the downregulation of p53, leads to a reduction in apoptosis rates in cardiac and vascular fibroblasts. The most pronounced beneficial effects on the cardiovascular system appear to be mediated by an enhancement of AMPK activity (phosphorylation). This activity induces a favorable increase in NO levels via the SIRT/Nrf2/eNOS pathway, contributing to the restoration of cellular oxidative balance. This outcome is also associated with enhanced activity of KLF-2, and consequently, increased expression of HO-1, GPx, and SOD alongside a decrease in iNOS expression. Furthermore, the increase in NO results in an elevation of cGMP, promoting vasodilation. Additionally, AMPK phosphorylation is involved in the downregulation of endothelial activation markers, platelet activation, and leukocyte adhesion, including ICAM, VCAM, and P-selectin, through the reduced expression of NOX2. Receptor–ligand binding also leads to the normalization of the balance between MMP-1 and MMP-2 and their inhibitors (TIMP-1 and TIMP-2), which contributes to the stabilization of atherosclerotic plaques. This process is likely mediated by AMPK as well. A crucial aspect of GLP-1/GLP-1 RA action is the inhibition of the RAGE/Rho/Rock-2 pathway, mediated not only by AMPK phosphorylation but also by the enhancement of cAMP/PKA activity. This leads to a reduction in the expression of NF-κB and its downstream inflammatory signaling pathways, resulting in diminished expression of proinflammatory cytokines such as IL-6, MCP-1, TNF-α, and additionally, ET-1, which is known to activate the RAGE/Rho/Rock-2 pathway. Consequently, this results in a significant reduction in the inflammation, which was also observed in patients. Moreover, receptor activation contributes to a reduction in the expression of TLR2 and TLR4, further attenuating the JNK-1/c-Jun signaling pathway, thereby limiting inflammation. Collectively, these mechanisms promote improvements in endothelial function and lipid profiles, which clinically correlate with the observed reduction in systemic inflammation, decreased risk of atherosclerosis, and, as demonstrated in clinical trials, a reduction in CVD risk and a decrease in MACE. Molecular changes highlighted in purple have also been reported in clinical studies. ↓—downregulation/decreased expression; ↑—upregulation/increased expression; ?—possible pathway.

The key molecular mechanisms, along with the associated molecular and clinical outcomes of GLP-1 RA and DPP-4i treatment that contribute to reduced atherosclerosis and CVD risk, are shown in Figure 1.

**Table 2:** Molecular and biochemical effects of GLP-1 RAs and DPP-4 inhibitors influencing cardiovascular health in studies related to atherosclerosis and coronary artery disease.

Source	Effect	Treatment	Model (Species, Intervention: Analyzed Tissues/Cell Line)
Preclinical in vivo studies			
Mice with experimental arterial hypertension: blood, heart, and vascular tissue analysis.	liraglutide 30 µg/kg twice daily i.p. for a week.	↓oxidative stress; ↓S-glutathionylation (a marker of eNOS uncoupling); ↑NO bioavailability.	[81]
Mice (apoE−/−, LDLr−/−) fed a Western diet: aorta and blood analysis.	liraglutide 1 mg/kg or semaglutide 4.0–60.0 μg/kg for 12–14 weeks (apoE−/−) or for 17 weeks (LDLr−/−).	↓TNF-α, IFN-γ, osteopontin, Il-6, Il-1RN, CCL2, OPN; ↓SELE, VCAM1; ↓MMP3, MMp13; ↓Cd163; ↓ABCA1, PTGIS; ↓total cholesterol; ↓triglyceride; ↓multiple inflammatory pathways.	[82]
Mice and rats treated with LPS: cardiac tissue and blood analysis.	linagliptin 5 mg/kg/day or liraglutide 200 µg/kg/day orally or s.c. for 3 days prior to and 6 h post LPS treatment.	↓Hb-NO levels and iNOS activity; ↓monocyte/macrophage infiltration; ↓expression of adhesion molecules VCAM-1, ICAM-1; ↓IL-6, MCP-1, and TNF-α; ↓aortic reactive oxygen species formation.	[88]
Mice, LDL receptor-deficient: blood and vascular tissue analysis.	Mice: liraglutide 300 mg/kg twice daily for 4 weeks.	In vivo: ↓LOX-1 expression in aortas; ↓plasma MDA; ↑ plasma SOD, NO; ↓plasma IL-1 beta, IL-6.	[89]
Rats fed a fructose-rich diet: analysis of the aorta and blood.	sitagliptin 5.0/10 mg/kg or exenatide 5/10 µg/kg for 4 weeks.	Exenatide: ↑plasma platelet-activating factor acetylhydrolase activity; ↓circulating ox-LDL and MCP-1; ↓PC/lyso-PC ratio; ↑apoB/apoA-I balance; ↓LDL oxidation; Sitagliptin: no change.	[95]
Mice with apoE knockout (apoE−/−): blood, heart, and aorta analysis.	Mice: liraglutide 300 µg/kg twice daily for 4 weeks.	↓PAI-1 and VAM expression; ↑endothelial nitric oxide synthase (eNOS); ↓ICAM-1 in aortic endothelium.	[106]
Rat diabetes mellitus model: cardiac tissue and blood analysis.	Rats: vildagliptin 1 mg/kg or exenatide 1 nmol/kg for 12 weeks.	In vivo: ↑glucose uptake and microvascular barrier function.	[108]
Rat, euglycemic insulin clamp after 2 weeks of high-fat diet: aorta tissue analysis.	liraglutide 200 μg/kg s.c. twice daily for 2 weeks.	↑ Nrf2 nuclear translocation; ↑AMPK phosphorylation; ↑muscle insulin sensitivity; ↑insulin-mediated muscle microvascular perfusion; ↓perivascular macrophage accumulation; ↓oxidative stress and inflammation.	[109]
Mice, apolipoprotein E–E-deficient: heart and aorta tissue and macrophage analysis.	Mice: Ex-4 300 pmol/kg/day or 24 pmol/kg/day.	↓TNF-α and MCP-1 levels; ↓nuclear translocation of p65; ↓NF-κB; ↓expression of CD11b.	[123]
Mice, wire-mediated endovascular injury: femoral artery analysis.	Mice: Ex-4 24 nmol/kg/day for 4 weeks.	↓SMC proliferation via the cAMP/PKA pathway; ↓TNF-α production by peritoneal macrophages in response to inflammatory stimuli.	[124]
Rat diabetes model: aorta tissue and blood analysis.	sitagliptin 30 mg/kg/day or exenatide 3 μg/kg/12 h for 12 weeks.	↑NO level in serum; ↓ET-1 level; ↓inflammatory cytokines levels; ↓oxidative stress; ↓expression level in AGE/RAGE-induced RhoA/ROCK/NF-κB/IκBα signaling pathways; ↑AMPK activation; ↓RAGE, ROCK-2, ET-1, iNOS, p-NF-κB, NF-κB, p-IκBα, and IL-6 expression restoring GLP-1R, p-eNOS, and p-AMPK expression.	[113]
Experimental in vitro studies			
HUVECs (ox-LDL-challenged human umbilical vein endothelial cells).	HUVECs: 1000 nM liraglutide for 1 h.	↓reactive oxygen species production; ↓apoptosis; ↓ICAM-1, VCAM-1, LOX-1, NOX4, and NF-κB expression.	[89]
Human umbilical vein endothelial cell line (C11-STH20).	C11-STH^20^: liraglutide 100 nM for 16 h.	↓TNF-α-mediated NF-κB induction, independent of PKA signaling.	[106]
Microvascular endothelial cells (CMECs).	CMECs: high glucose medium, GLP-1 (10^−10^, 10^−9^, 10^−8^, 10^−7^ mol/L).	↓reactive oxygen species production; ↓apoptotic index; ↓levels of NADPH oxidases; ↑cAMP/PKA; ↓Rho expression.	[108]
Mouse macrophage cell line RAW264 and mouse preadipocyte cell line 3T3-L1s.	Ex-4 2.5 nM.	↓TNF-β, IL-6, and IL-1β secretion; ↓activation of NF-κB in macrophages.	[116]
Rat vascular smooth muscle cells (VSMCs).	liraglutide 100 nM for 1h.	↓apoptosis; ↑phosphorylation of protein kinase B (Akt) and ERK1/2.	[121]
THP-1 cell line.	THP-1: Ex-4 0.03, 0.3, and 3 nmol/L for 1 h.	↓TNF-α and MCP-1 levels; ↓nuclear translocation of p65; ↓NF-κB; ↓expression of CD11b.	[123]
Rat aortic SMCs.	rat aortic SMCs: Ex-4 5 nmol/L, 60 min.	↓SMC proliferation via the cAMP/PKA pathway.	[124]
Human primary endothelial cells, coronary artery endothelial cells, and aorta endothelial cells.	exenatide 1 and 10 nmol/L for 24 h.	↓caspase 3/7 activation; ↓NF-kB activation; ↓GFP expression; ↓VCAM-1 and ICAM-1 expression; ↑TIMP-1 and TIMP-2.	[93]
Human aortic endothelial cells (HAECs), ox-LDL-treated.	dulaglutide 50 and 100 nM for 24 h.	↓ox-LDL-induced oxidative stress and mitochondrial dysfunction; ↓secretion of IL-1β, IL-6, MCP-1, and HMG-1; ↓cell viability and release of LDH; ↓attachment of THP-1 to HAECs by inhibiting VCAM-1, E-selectin; ↑expression of KLF2 through inhibiting the activation of p53.	[125]
Human studies			
Human women with PCOS: blood analysis.	liraglutide in increasing doses (0.6–1.8 mg) s.c. for six months.	↓HOMA-IR; ↓triglyceride; ↓hsCRP; ↓urinary isoprostanes; ↓sP-selectin, sICAM, and sVCAM; ↓platelet P-selectin expression.	[79]
Humans with type 2 diabetes: blood and serum analysis.	GLP-1 RA treatment for one year.	↓ROS production; ↑mitochondrial membrane potential, oxygen consumption; ↓MPO; ↓ICAM-1, VCAM-1, IL-6, TNF-α, and IL-12; ↑IL-10; ↓CIMT.	[80]
Humans with type 2 diabetes: blood and serum analysis.	liraglutide 0.6 mg once daily for 2 weeks and increasing to 1.2 mg once daily.	↓TNF-α; ↓IL-1β; ↓IL-6; ↑adipokine adiponectin.	[111]
Humans with type 2 diabetes: blood, serum, and urine analysis.	liraglutide 1.2–1.8 mg/day for 26 weeks.	↓IL-6 ↓albumin/creatinine ratio in urine.	[117]
Humans with type 2 diabetes and albuminuria: blood and serum analysis.	liraglutide 1.8 mg/day for 12 weeks.	↓TNF-α level; ↓MR-proADM level; ↓MR-proANP level.	[118]
Humans with type 2 diabetes and obesity: blood and serum analysis.	exenatide 10 μg s.c. twice daily for 12 weeks.	↓blood glucose; ↓HbA1c; ↓free fatty acids; ↓reactive oxygen species generation; ↓NF-κB; ↓mRNA expression of TNF-α, IL-1β, JNK-1, TLR-2, TLR-4, and SOCS-3 in mononuclear cells; ↓monocyte chemoattractant protein-1; ↓MMP9; ↓serum amyloid A; ↓IL-6.	[119]
Humans with type 2 diabetes: blood and serum analysis.	sitagliptin 100 mg/day for 12 weeks.	↓HbA1c; ↑glucagon-like peptide-1 concentrations; ↓expression of CD26, TNF-α, TLR-4, TLR-2, c-Jun N-terminal kinase-1, and inhibitory-κB kinase (IKKβ) (proinflammatory kinases) in mononuclear cells; ↓expression of TLR-2, IKKβ, CCR-2, and CD26 and NF-κB; ↓expression of JNK-1, IKKβ, and TLR-4 and plasma concentrations of C-reactive protein, IL-6, and free fatty acids.	[120]

↓ indicates lowered/downregulated expression/concentration/activity; ↑ indicates increased/upregulated expression/concentration/activity. s.c.: subcutaneous administration; i.p.: intraperitoneal administration.

### 2.4. Cardiac Arrhythmias

Current evidence does not clearly support a significant beneficial effect of GLP-1 RA in the treatment of cardiac arrhythmias. A high-quality meta-analysis of randomized controlled trials by Boulmpou et al. found that GLP-1 RA treatment did not significantly reduce the risk of atrial fibrillation (AF), atrial flutter, ventricular fibrillation, ventricular tachycardia, sinus node dysfunction, ventricular extrasystoles, second-degree atrioventricular block, or complete atrioventricular block [126]. Although cardiovascular benefit has been observed in obese patients without diabetes, meta-analyses of randomized controlled trials did not show significant differences regarding the incidence of arrhythmia, including AF, in patients treated with GLP-1 RA [74,75]. Similarly, large real-world cohort studies by Chan et al. found no difference in the risk of new-onset AF between patients treated with DPP-4i and those using GLP-1 RA [127,128].

A meta-analysis by Zhong et al. also found that liraglutide did not improve left ventricular systolic or diastolic function. No significant differences were observed in left ventricular ejection fraction (LVEF) at the end of the study, in ΔLVEF over the study duration, or in the ratio of mitral annular early diastolic velocity to early diastolic filling velocity [129].

Although GLP-1 RAs appear to have a neutral effect on cardiac arrhythmias, a pharmacovigilance analysis using the FDA Adverse Event Reporting System (FAERS) by Thotamgari et al. indicated that linagliptin was associated with the highest proportion of AF events in diabetic patients. Saxagliptin, liraglutide, sitagliptin, semaglutide, and exenatide contributed to fewer AF incidents. The proportion of cardiac adverse events attributable to AF was highest for liraglutide, followed by semaglutide, exenatide, linagliptin, and dulaglutide. Notably, in the FAERS database, only DPP-4i showed a signal of disproportionate reporting for AF, whereas no such signal was observed for GLP-1 RA [130].

The available evidence suggests that GLP-1 RA has a neutral effect on cardiac arrhythmias. This conclusion mainly comes from meta-analyses of randomized controlled trials and large observational studies. While pharmacovigilance data indicate possible signals for some agents, these findings should be interpreted carefully and do not prove causation. This highlights the need for more prospective studies that focus specifically on arrhythmic outcomes.

#### 2.4.1. Molecular Mechanisms Involved in GLP-1 RA and DPP-4i Effects in Cardiac Arrhythmias

Despite the lack of clinical evidence for the beneficial effects of GLP-1 RA and DPP-4i in cardiac arrhythmias, a few experimental studies related to the mechanisms of cardiac electrophysiology and structural remodeling were undertaken.

##### Cardiac Remodeling and Fibrosis

Animal studies suggest that GLP-1 RA may influence cardiac remodeling and fibrosis [131]. In a study by Wang et al., liraglutide was found to inhibit proliferation, migration, and ECM deposition in angiotensin II (AngII)-stimulated mouse atrial fibroblasts as well as the invasion of mouse fibroblast cells [132]. The inhibition of fibroblast migration by liraglutide was mediated through the microRNA-21/phosphatase and tensin homolog/phosphoinositide 3-kinase (miR-21/PTEN/PI3K) signaling pathway. This axis is critically involved in fibroblast activation and cardiac fibrosis [132]. Liraglutide suppressed AngII-induced upregulation of miR-21 [133] while increasing PTEN expression and inhibiting the PI3K/AKT signaling pathway [132].

Wójcicka et al. reported that exenatide administration reduced fructose-induced increases in plasma levels of asymmetric dimethylarginine (ADMA), an endogenous NOS inhibitor; however, its levels in the heart remained unchanged. Despite this, elevated NO and PRMT levels, reduced TGF-ß1 and α-SMA levels, and decreased COL1A1 expression all suggested a reduction in fibrosis in the cardiac tissue. Exenatide and sitagliptin also downregulated the immunoexpression of Smad2/3/P. These proteins induce fibroblast-to-myofibroblast conversion and collagen production, leading to myocardial and perivascular fibrosis. In metabolic syndrome-induced rats, both sitagliptin and exenatide positively modulated cardiac fibrotic remodeling, although the process was ADMA-independent [134].

AF is one of the most common arrhythmias [135,136]. Its primary pathophysiological hallmark is cardiac fibrosis, which involves abnormal fibroblast activation, proliferation, and differentiation, leading to the accumulation of fibrillar and non-fibrillar collagen [137]. Untreated arrhythmias, especially atrial fibrillation, can cause serious problems such as blood clots, heart failure, or sudden cardiac death [138,139]. While GLP-1 RAs appear to have a neutral effect on clinical outcomes in patients with AF, some concerns have been raised regarding a potential increased risk of AF with DPP-4is in observational studies. Nonetheless, the positive molecular effects observed in preclinical studies suggest that GLP-1 RAs, particularly liraglutide and exenatide, may exert beneficial anti-fibrotic and metabolic effects that could be relevant in AF. These early findings suggest that GLP-1 RAs could be explored further as potential factors in cardiac remodeling.

The role of the miR-21/PTEN/PI3K signaling pathway in GLP-1 RA effects on heart remodeling offers a promising area for more study. Further translational and clinical research is required to confirm these findings.

The molecular and biochemical effects of GLP-1 RAs and DPP-4is have been summarized in Table 3.

**Table 3:** Molecular and biochemical effects of GLP-1 RA and DPP-4i treatment influencing cardiovascular health in the research concerning cardiac arrhythmias.

Source	Effect	Treatment	Model (Species, Intervention: Analyzed Tissues)
Preclinical in vivo studies			
Mice, angiotensin II-induced proliferation model (atrial fibrillation model): atrial fibroblast analysis.	Liraglutide 10, 50, or 100 nmol/L.	↓AngII-induced increase in the expression level of miR-21; ↑expression of PTEN (a target of miR-21); ↓phosphoinositide 3-kinase (PI3K)/AKT signaling pathway; ↓angiotensin II-induced proliferation, migration, and invasion of fibroblasts.	[132]
Rats fed a fructose-rich diet: analysis of fibrosis, aorta, and blood.	Sitagliptin 5.0/10 mg/kg or exenatide 5/10 µg/kg for 4 weeks.	Exenatide: ↓ADMA (plasma); ↑NO and PRMT; ↓TGF-ß1 and α-SMA; ↓COL1A1 expression; Sitagliptin: ↑NO; ↓circulating SDMA; ↑renal DDAH activity; ↓myocardial DDAH activity; Exenatide and sitagliptin: ↓immunoexpression of Smad2/3/P proteins; positively modulated cardiac fibrotic remodeling.	[134]

↓ indicates lowered/downregulated expression/concentration/activity; ↑ indicates increased/upregulated expression/concentration/activity.

### 2.5. Myocardial Infarction and Heart Failure

Patients with type 2 diabetes (T2DM) face an increased risk of developing heart failure (HF) [140]. Other major risk factors include obesity [141] and hypertension [142]. Approximately 50% of individuals with HF also have T2DM [143]. A narrative review by Kreiner et al. indicated that the use of GLP-1 RAs has been shown to significantly improve the quality of life in HF patients [144]. Moreover, the treatment might reduce cardiovascular risk in T2DM patients and is recommended for lowering the risk of myocardial infarction (MI) with potential benefits in reducing HF-related hospitalizations [145].

A retrospective cohort analysis by Riley et al. [73] demonstrated that GLP-1 RA therapy significantly lowered the risk of major cardiovascular events, including acute MI and HF, in T2DM patients over five years compared with those receiving insulin alone. As an observational study, these results should be interpreted with caution.

In a double-blind, randomized, placebo-controlled, event-driven superiority trial, semaglutide was associated with a reduced risk of MI in overweight and obese individuals [146]. Also, a previous meta-analysis by Monami et al. found that GLP-1 RAs, including lixisenatide, liraglutide, dulaglutide, semaglutide, exenatide, and albiglutide, reduced cardiovascular mortality and the incidence of MI in T2DM patients compared with placebo or other non-GLP-1 RA treatments. However, in this study, GLP-1 RAs did not significantly impact the overall incidence of HF [147].

In contrast, several investigations have raised concerns regarding the safety of DPP-4i treatment. The results of two large clinical trials involving patients with heart disease, SAVOR-TIMI 53 (saxagliptin) [148] and EXAMINE (alogliptin) [149,150], showed that hospitalization for HF occurred in 3.5% of saxagliptin users and 3.9% of alogliptin users (compared with 2.8 and 3.3% in the placebo groups, respectively) [151]. These findings led the FDA to issue warnings regarding the risk of HF for these drugs. However, the same studies found that saxagliptin did not significantly affect other cardiovascular outcomes, including coronary revascularization, MI, cardiovascular mortality, hospitalization for unstable angina, or HF [148]. Similarly, treatment with alogliptin in T2DM patients with recent acute coronary syndrome did not increase the risk of cardiovascular death or nonfatal MI compared with placebo [149,150].

However, further clinical trials with another DPP-4i have not found an increased risk of HF in patients using vildagliptin [67,152]. Moreover, the CARMELINA and CAROLINA trials concluded that linagliptin had a neutral effect on cardiovascular risk in T2DM patients with a high risk of cardiovascular events [153,154]. Similarly, the TECOS clinical trial [155] reported that sitagliptin neither increased nor significantly reduced cardiovascular risk in T2DM patients with established cardiovascular disease. Furthermore, the study found no elevated risk of HF-related hospitalizations [155].

Neuen et al. additionally described the benefits of combination therapy with GLP-1 RAs, SGLT2is, and the nonsteroidal mineralocorticoid receptor antagonist (ns-MRA) finerenone in patients with albuminuria, T2DM, and cardiovascular disease. Their model estimated that a 50-year-old patient with these conditions, without cardiovascular events and untreated, would have a life expectancy of 17.9 years, whereas combination therapy could extend survival to 21.1 years [156].

GLP-1 RAs were shown to reduce mortality and major cardiovascular events more effectively than sulfonylureas or DPP-4is in patients with established or high-risk cardiovascular disease in a meta-analysis by Brønden et al. [157]. A small randomized, double-blind, crossover study suggested that vildagliptin may improve endothelium-dependent vasodilation in T2DM patients [158]. Furthermore, in an exploratory subgroup analysis within an RCT meta-analysis, it was hypothesized that vildagliptin’s positive effects on systolic blood pressure, LDL cholesterol, hypoglycemia, and weight—observed in T2DM patients under 65 years old but not in those over 65—may contribute to a lower risk of MACE in younger patients [66]. However, one randomized placebo-controlled trial suggested that vildagliptin might lead to increased left ventricular volumes of unknown clinical significance [159]. Additionally, vildagliptin use may be associated with an increased risk of angioedema in patients taking ACE inhibitors according to a meta-analysis by Brown et al., though the absolute risk remains low [160]. Despite the low risk, GLP-1 RAs might be considered a safer choice than DPP-4is.

Lyu et al. found that GLP-1 RAs were associated with a lower risk of hospitalization due to cardiovascular disease in T2DM patients compared with DPP-4is. While overall hospitalization rates for all causes and CVD-related events were similar between GLP-1 RAs and SGLT2is, the latter were linked to a lower risk of HF-related hospitalization [161]. GLP-1 RAs outperform DPP-4is in reducing cardiovascular and renal complications [162].

Despite their benefits, GLP-1 RAs are often underprescribed in frail and elderly patients due to concerns about adverse effects such as diabetic ketoacidosis, dehydration, and urinary tract infections. However, studies indicate that frail individuals with multimorbidity and polypharmacy derive the greatest absolute benefits from these treatments [163], suggesting that their use in older, non-malnourished patients with appropriate indications is justified [164]. Researchers recommend GLP-1 RAs for HF patients with preserved ejection fraction (HFpEF) due to their potential to reduce cardiovascular events. However, they are not recommended for patients with reduced ejection fraction (HFrEF), as GLP-1 RAs may increase arrhythmia risk and HF-related events in this group [145].

#### 2.5.1. Molecular Mechanisms Involved in GLP-1 RA and DPP-4i Effects in Myocardial Infarction and Heart Failure

##### Natriuretic Peptides and Extracellular Matrix Remodeling

Semaglutide may play a protective role in preventing cardiac tissue damage following MI. In a preclinical study using an animal model of myocardial ischemia/reperfusion (I/R) injury, semaglutide increased GLP-1R expression, activating the PKG/PKCε/ERK1/2 (protein kinase G/protein kinase C epsilon/extracellular signal-regulated kinase 1/2 pathway, which inhibited cardiomyocyte apoptosis. Additionally, it reduced hs-cTNT levels and increased NT-proBNP levels, findings that may provide a basis for further investigation into the treatment of myocardial I/R injury [165]. Moreover, GLP-1 RAs have been shown to reduce monocyte/macrophage infiltration in cardiac tissue, further supporting their cardioprotective potential [88].

In heart failure, the release of atrial natriuretic peptide (ANP) and brain natriuretic peptide (BNP) increases due to ventricular myocyte stimulation in response to elevated norepinephrine and angiotensin levels as well as heightened ventricular filling pressures [166,167]. As shown in a cross-sectional observational clinical study, patients with acute coronary syndrome (ACS) exhibited increased levels of natriuretic peptides, including ANP, while endogenous GLP-1 levels were reduced, suggesting an opposing role of GLP-1 and ANP in cardiovascular homeostasis [168]. In a small clinical study, individuals with T2DM and heart failure with HFrEF on prolonged liraglutide therapy were shown to decrease NT-proBNP and MR-proANP levels while improving clinical outcomes [169]. Since liraglutide enhances GLP-1R expression and activates the PI3K/AKT/mTOR pathway, it has been proposed that the drug may suppress ANP secretion and improve cardiac function through this mechanism. These effects are GLP-1R-dependent, as they were abolished in the presence of a GLP-1 receptor antagonist [168]. Additionally, improvements in NT-proBNP levels may result from reduced oxidative stress in newly diagnosed T2DM patients [170].

Improved cardiomyocyte function is also mediated by DPP-4is. Sitagliptin was found to improve passive left ventricular compliance, increase endothelial cell density, reduce myocyte hypertrophy, and decrease collagen 1 abundance in an in vivo study involving diabetic rats following MI [171], suggesting that DPP-4 inhibitors may attenuate aspects of cardiac dysfunction and adverse remodeling after MI. In contrast, vildagliptin did not reverse cardiac remodeling in an MI model, nor did it significantly reduce ANP and BNP mRNA levels or alter cardiomyocyte size or capillary density in non-diabetic animals [172]. However, no adverse effects of vildagliptin were observed. Additionally, in a multicenter, open-label, parallel-group comparison clinical study, vildagliptin did not improve systolic or diastolic cardiac function six months after MI, although BNP levels were reduced [173]. While vildagliptin was associated with improved endothelial function and endothelium-dependent vasodilation in T2DM patients [158], its use was also linked to increased left ventricular volumes [159]. However, the clinical relevance of this finding remains uncertain and warrants further study [159].

##### Mitochondrial Function

Heart failure progression is strongly associated with impaired mitochondrial dynamics [174]. Dulaglutide treatment was shown to limit cardiac remodeling and dysfunction while supporting myocardial energy metabolism in the diabetic mouse model [175]. It also protected against T2DM-induced cardiac lipotoxicity [175]. The cardioprotective effects of GLP-1 RAs might result, at least in part, from their antioxidant and anti-inflammatory properties as well as their ability to enhance mitochondrial function. Dulaglutide was shown to reduce mitochondrial fragmentation in cardiomyocytes, restoring normal morphology and function in the T2DM mouse model. Additionally, in those preclinical studies, dulaglutide maintained mitochondrial homeostasis by enhancing AMPK alpha 2 (AMPKα2) signaling [175]. Notably, dulaglutide upregulated phosphorylated (Ser 637) Drp-1 while reducing total dynamin-related protein-1 (Drp-1) protein levels [175]. This phosphorylation inhibits Drp-1 GTPase activity, thereby suppressing mitochondrial fission [176]. Excessive mitochondrial fission and depolarization can trigger collagen expression through forkhead box protein O1 (FoxO1) activation, a transcription factor that regulates COX-2 gene expression in cardiac fibroblasts, leading to fibrosis [177]. By inhibiting mitochondrial fission in diabetic cardiomyocytes, dulaglutide may help mitigate cardiac remodeling and fibrosis in patients with chronic heart failure, though further validation in clinical settings is needed [175]. Additionally, Drp-1 inhibition has been linked to reduced hypertensive cardiac hypertrophy [178].

Another drug, liraglutide, increased blood pressure, shortened QRS duration, and reduced oxidative stress levels in aged rats. As preclinical studies indicated, it also improved potassium (K^+^) channel function and calcium homeostasis, likely through enhanced Na^+^/Ca^2+^ exchanger currents. In isolated cardiomyocytes, liraglutide restored normal mitochondrial membrane potential in vitro. Interestingly, liraglutide administration was associated with SGLT2 downregulation, increased insulin receptor substrate-1 (IRS1) expression, and decreased protein kinase G (PKG) activity. It also improved the phosphorylated nitric oxide synthase 3/nitric oxide synthase 3 (pNOS3/NOS3) ratio, suggesting a protective effect against oxidative stress in aging cardiac tissue via the IRS1-eNOS-PKG pathway [81,179].

##### Myocardial Damage

GLP-1 RAs have demonstrated greater efficacy in cardiovascular protection compared with DPP-4 inhibitors, underscoring the role of GLP-1 metabolites in cardioprotection. In a preclinical model, Ban et al. showed that not only GLP-1 (7–36) but also GLP-1 (9–36) contributed to these effects, suggesting that the overall cardioprotective action is not entirely mediated by GLP-1 receptors. In ex vivo studies, GLP-1 administration improved functional recovery and cardiomyocyte viability after I/R injury in isolated hearts, and these benefits were observed even in GLP-1R knockout mice. Although GLP-1 (9–36) had only a moderate effect on glucose uptake and inotropic response in myocardial tissue, administration of both the full-length and truncated peptides, but not the DPP-4-resistant exendin-4, reduced ischemic damage and increased cGMP/NO release. This led to vasodilation and improved coronary blood flow, even in mice lacking GLP-1 receptor expression [24]. These findings underscore the complexity of GLP-1 signaling in cardiovascular tissues.

In preclinical studies, exenatide was also shown to limit myocardial injury and improve heart function in donation after circulatory death (DCD) hearts. In juvenile pigs treated with exenatide during reperfusion, myocardial oxygen consumption increased, troponin-I release decreased, diastolic function improved, lactate levels were lower, eNOS activation was enhanced, and endothelial damage was reduced [180]. These findings further support the role of GLP-1 RAs in cardioprotection, particularly in the context of ischemic injury and post-MI remodeling. Nevertheless, these effects were observed in the context of short-term reperfusion, and long-term functional outcomes were not evaluated [180].

Current clinical evidence supports the use of GLP-1 receptor agonists (GLP-1 RAs) in reducing cardiovascular events, including myocardial infarction, particularly in patients with type 2 diabetes and high cardiovascular risk. Although their effects on heart failure outcomes remain neutral or modest, preclinical data suggest that GLP-1 RAs exert pleiotropic cardioprotective effects through the modulation of natriuretic peptide levels, suppression of cardiomyocyte apoptosis, inhibition of extracellular matrix remodeling, and enhancement of mitochondrial dynamics. In addition, GLP-1 RA treatments have been shown to improve blood–brain barrier integrity, attenuate systemic inflammation, and enhance cardiac function, indicating their potential to limit ischemic damage and adverse post-myocardial infarction (MI) remodeling. Compared with DPP-4 inhibitors, GLP-1 RAs exhibit superior efficacy in cardiovascular protection, with GLP-1 metabolites contributing to improved functional recovery and cardiomyocyte viability following ischemia/reperfusion injury. However, the majority of these mechanistic insights originate from preclinical models. Robust translational studies are needed to validate these mechanisms in humans. The molecular and biochemical effects of GLP-1 RAs and DPP-4is have been summarized in Table 4.

**Figure 2:**
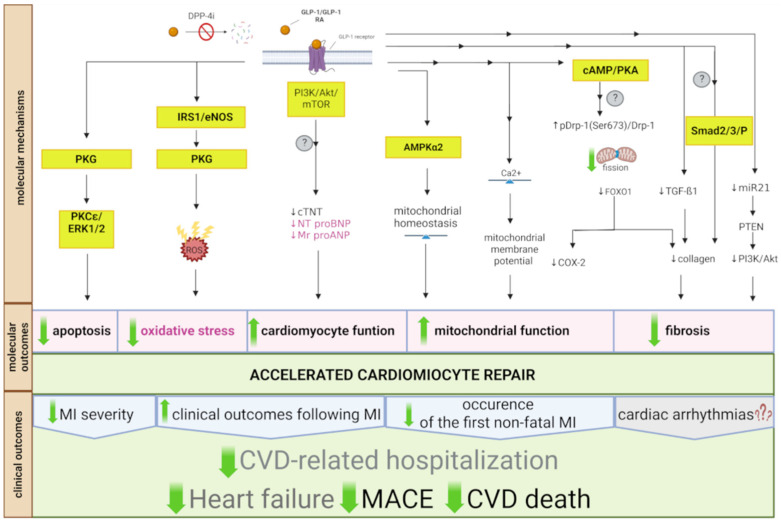
The key molecular mechanisms along with the associated molecular and clinical outcomes of GLP-1 RA and DPP-4i treatments that contribute to decreased heart failure and CVD-related deaths. GLP-1 receptor agonists (GLP-1 RAs) bind to the GLP-1 receptor, a member of the G protein-coupled receptor family. The use of DPP-4 inhibitors (DPP-4is) reduces the activity of the enzymes responsible for the degradation of GLP-1. After receptor engagement, activation of the PKG/PKCε/ERK1/2 signaling pathway protects cells from apoptosis, while the IRS1/eNOS/PKG pathway contributes to a reduction in oxidative stress in experimental myocardial infarction (MI) models. Patients treated with GLP-1 RAs exhibit decreased circulating levels of NT-proBNP and Mr-proANP heart failure markers, which has also been observed in experimental models alongside a reduction in cTnT levels. This process is most likely regulated by the activity of the PI3K/Akt/mTOR pathway. The use of GLP-1 RA and DPP-4i is also associated with improved mitochondrial function due to the maintenance of calcium homeostasis of mitochondrial membrane potential. Through AMPKα2 activation, mitochondrial homeostasis is maintained, and increased phosphorylation of Drp-1 (Ser673), likely mediated by the cAMP/PKA pathway, reduces mitochondrial fission, decreasing the expression of COX-2 and collagen. As a result, a reduction in fibrosis is observed. This process is also mediated by decreased TGF-β1 as well as, in experimental models, a reduction in cardiac arrhythmias via the downregulation of the miR21/PTEN/PI3K/Akt pathway and Smad2/3/P. In experimental MI models, these molecular effects facilitate faster tissue regeneration after cardiac incidents and decrease their severity. Clinically, patients treated with GLP-1 RAs show improved outcomes following MI; a reduction in the occurrence of first nonfatal MI, which is associated with an observational decrease in hospitalization rates related to CVD and heart failure (gray); and a clinical reduction in MACE occurrence and CVD-related deaths. Despite promising results in in vivo studies using models mimicking cardiac arrhythmias, these effects have not yet been clearly linked to clinical outcomes. ↓—downregulation/decreased expression; ↑—upregulation/increased expression; ?—possible pathway.

The key molecular mechanisms, along with the associated molecular and clinical outcomes of GLP-1 RA and DPP-4i treatment that contribute to decreased heart failure and CVD-related deaths, are shown in Figure 2.

**Table 4:** Molecular and biochemical effects of GLP-1 RA and DPP-4 inhibitor treatments influencing cardiovascular health in the research models associated with myocardial infarction (MI) and heart failure.

Model (Species, Intervention: Analyzed Tissues)	Treatment	Effect	Source
Preclinical in vivo studies			
Mice, ischemia/reperfusion (MI) model: cardiac tissue analysis.	5 nmol exenatide during reperfusion.	↑cGMP/NO release; ↑glucose uptake; ↑cAMP and cGMP release.	[24]
Rats, ischemia/reperfusion injury (MI): cardiac tissue analysis.	semaglutide 0.3 mg/kg, 30 min prior to ischemia/reperfusion injury.	↑GLP-1R expression; ↑PKG/PKCε/ERK1/2 pathway; ↓cardiomyocyte apoptosis; ↓hs-cTNT levels; ↑NT-proBNP levels.	[165]
Rats, isolated atrial perfusion model: atrial tissue analysis. Mice, DPP-4 knockout (DPP-4−/−): cardiac tissue and blood analysis.	Mice: liraglutide 30 μg/kg/day for 14 days; Rats, ex vivo: liraglutide 3.2 nmol/L.	↓ANP secretion; ↑expression of GLP-1R and PI3K/AKT/mTOR.	[168]
Rats, MI model: blood and cardiac tissue analysis.	sitagliptin 300 mg/kg/day via oral tube for 6 weeks.	↓collagen 1 abundance.	[171]
Rats, MI model: cardiac tissue analysis.	pretreatment with vildagliptin 15 mg/kg/day for 2 days or 3 weeks after coronary artery ligation.	No reduction in ANP and BNP mRNA levels.	[172]
Mice, high-fat diet/streptozotocin-induced type 2 diabetes model: cardiac tissue and blood analysis.	dulaglutide 0.6 mg/kg/week s.c. for 8 weeks.	↑AMPKα2 signaling; ↓mitochondrial fragmentation in cardiomyocytes; ↓insulin resistance; ↑ glucose tolerance; ↓hyperlipidemia; ↑fatty acid use in the myocardium; ↑phosphorylated (Ser 637) Drp-1 while ↓total Drp-1 protein levels.	[175]
Rats, aged (24 months) and adult (6 months): freshly isolated ventricular cardiomyocytes.	Adult rats: liraglutide 200 µg/kg/day i.p. for 6 weeks; Aged rats: liraglutide 300 µg/kg/day i.p. for 4 weeks.	↑blood pressure; ↓oxidative stress; ↑Na+/Ca^2+^ exchanger currents; ↓SGLT2; ↑IRS1 expression; ↓protein kinase G (PKG) activity; restored normal mitochondrial membrane potential; improved the pNOS3/NOS3 ratio.	[179]
Pigs after global warm ischemia/reperfusion (MI): cardiac tissue analysis.	exenatide 5 nmol during reperfusion.	↑myocardial oxygen consumption; ↑activated eNOS; ↓ troponin-I; ↓endothelial damage; ↓lactate.	[180]
Human studies			
Humans with heart failure with reduced ejection fraction, with/without type 2 diabetes: blood analysis.	liraglutide 1.8 mg/day for 24 weeks	↓NT-proBNP and MR-proANP levels; no effect on MR-proADM and copeptin.	[169]
Humans with type 2 diabetes mellitus: blood analysis.	liraglutide 1.8 mg/day s.c. for 6 months	↓malondialdehyde (oxidative stress indicator); ↓NT-proBNP.	[170]
Human, post-acute MI: blood analysis.	vildagliptin 50 mg twice a day for 6 months	↓BNP levels; ↓HbA1c levels.	[171]

↓ indicates lowered/downregulated expression/concentration/activity; ↑ indicates increased/upregulated expression/concentration/activity. MI: myocardial infarction; s.c.: subcutaneous administration; i.p.: intraperitoneal administration.

## 3. Conclusions

Our review is unique due to its comprehensive approach, encompassing and integrating the clinical advantages of GLP-1 RAs and DPP-4is and the intricate biochemical and molecular mechanisms underpinning these benefits.

CVD remains the leading cause of mortality, with one person dying every 33 s due to CVD-related complications [181]. Modern lifestyle factors such as nicotine and alcohol consumption, stress, sedentary behavior, and obesity contribute significantly to the rising prevalence of CVD [182]. There is an urgent need for therapies that address both metabolic and vascular dysfunction.

Type 2 diabetes, which frequently predisposes individuals to and coexists with CVD, has brought attention to the cardioprotective properties of certain antidiabetic medications. This includes the well-established benefits of SGLT2 inhibitors as well as the growing recognition of GLP-1 RAs and DPP-4 inhibitors in cardiovascular protection.

Compared with SGLT2 inhibitors, which are a primary treatment used during HF management, the GLP-1 RAs seem to have less of an impact on reducing HF-related hospitalizations. The use of SGLT2is in patients with overt cardiovascular disease has proven more efficient in the prevention of HF events. An Italian cohort study using GLP-1 RA and SGLT2i did not find any difference between the two groups with regard to HF-related hospitalization rates; however, other studies have shown favorably lower risk of congestive heart failure and cardiovascular events associated with SGLT2i use. It therefore appears that there is greater benefit provided by SGLT2is in the context of secondary prevention. Nonetheless, when high CVD risk coexists with obesity, the ADA indications are to use GLP-1 RAs rather than SGLT2is.

GLP-1 receptor agonists have shown consistent decreases in MACE, such as myocardial infarction and cardiovascular-related hospitalizations, in several large-scale clinical trials. The cardioprotective effects of GLP-1 RAs are largely mediated through their interaction with the GLP-1 receptor, which is highly expressed in the cardiovascular system [183].

Both GLP-1 receptor agonists and endogenous GLP-1 attenuate apoptotic processes and downregulate the expression of proinflammatory cytokines, counteract cardiac lipotoxicity, and mitigate oxidative stress within the cardiovascular system. Furthermore, they decrease markers of atherothrombosis and adhesion molecules, thereby improving endothelial function. In heart failure, GLP-1 RAs have been found to reduce cardiac tissue remodeling and fibrosis while enhancing mitochondrial function. Although many of these mechanisms have been uncovered in preclinical or translational studies, they strongly justify the clinical advantages observed. These effects likely underpin the substantial cardiovascular benefits associated with GLP-1 RA therapy, including reduced risk, severity, and complications of atherosclerosis [121], myocardial infarction, arrhythmias, heart failure, and MACE (Table 5). These benefits are reflected in lower hospitalization rates for cardiovascular-related complications among patients treated with GLP-1 RAs.

In contrast, DPP-4 inhibitors exhibit significantly lower efficacy in the prevention and management of cardiovascular diseases despite some evidence of modest improvements in vascular and endothelial function. This discrepancy suggests that the cardioprotective effects of GLP-1 may be attributed not only to GLP-1 (7–36) but also to its metabolite, GLP-1 (9–36).

The growing clinical utility of GLP-1 RAs encourages broader implementation in patients at heightened cardiovascular risk, further supporting their integration into routine cardiometabolic care. Current research endorses the use of GLP-1 RAs in the cardiometabolic management of patients with T2DM, obesity, and high cardiovascular risk, since these agents, in addition to metabolic control, address some of the important pathophysiological drivers of cardiovascular disease. However, more translational studies as well as long-term clinical trials are needed to fully clarify their efficacy and refine treatment approaches, especially in relation to or together with SGLT2 inhibitors, with emphasis on their role in both secondary and primary prevention.

Additionally, future studies should consider specific patient groups and populations, as they differ in their genetic backgrounds, which might influence drug responses and expected outcomes. This variability can significantly impact the effectiveness and safety of treatments. By tailoring research to these distinct groups, the results can be more accurately applied to clinical practice, ensuring the best possible outcomes. Such an approach will facilitate the development of personalized medicine strategies that optimize treatment efficacy and minimize adverse effects.

## 4. Materials and Methods

This comprehensive review was conducted in accordance with PRISMA reporting guidelines [184] and the recommendations outlined in the Cochrane Handbook for Systematic Reviews of Interventions [185].

### 4.1. Search Strategy

A comprehensive literature search was conducted using MedLine (PubMed) from 1990 to January 2025, including both free-text terms and combinations of MeSH subject headings. The researchers searched databases to find all relevant materials regarding the molecular perspective on the cardioprotective effects of GLP-1 receptor analogs and DPP-4 inhibitors. The final search strategy was refined through multiple pre-search trials to optimize sensitivity and specificity. The keywords used included, “Glucagon like peptide-1 receptor agonist cardiovascular”, “dipeptidyl peptidase-4 inhibitor cardiovascular outcomes”, “GLP-1 inflammation”, “Oxidative stress”, “Mitochondrial function”, and “Molecular mechanisms of GLP-1”.

### 4.2. Eligibility Criteria

**Figure 3:**
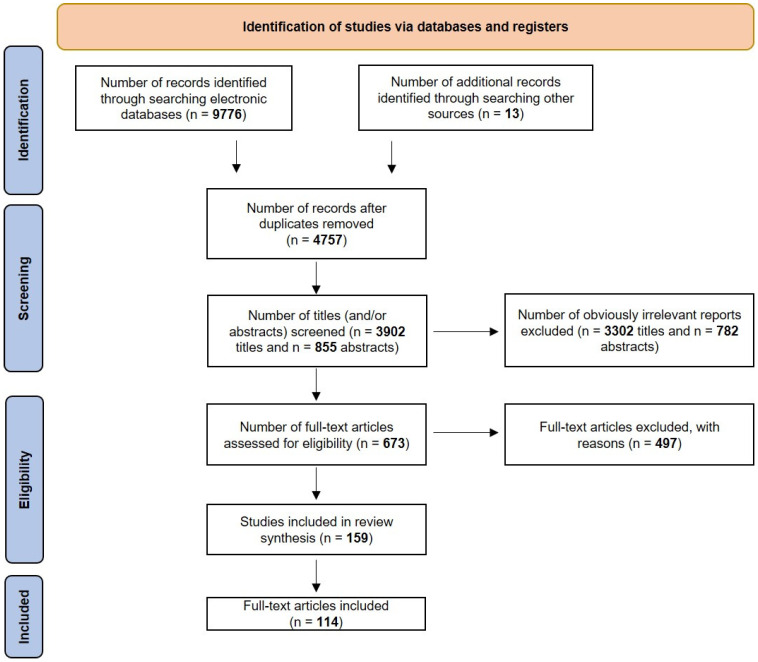
Summary of study search and selection.

Studies were selected based on the following inclusion criteria: (1) prospective clinical trials; (2) retrospective clinical trials; (3) in vivo and in vitro studies; (4) meta-analyses; and (5) studies published in English. Eligible articles were required to investigate the cardioprotective effects and therapeutic potential of GLP-1 RA and DPP-4i, with a particular focus on their molecular impact on the cardiovascular system. Exclusion criteria included (1) studies lacking robust statistical analysis; (2) papers addressing unrelated aspects of GLP-1 or/and DPP-4i treatment; (3) unpublished or non-peer-reviewed literature; and (4) articles on aspects of GLP-1 or/and DPP-4i treatment other than cardioprotection (Figure 3). Duplicate records were removed using the Zotero reference management software version 6.0.36 (Corporation for Digital Scholarship, Vienna, VA, USA). Two independent researchers (J.Ż. and M.K.) screened the remaining articles based on title and abstract, followed by a full-text review to ensure relevance. Any discrepancies in study selection were resolved through discussion or consultation with a third and fourth investigator (P.K. and P. Kapczuk).

## Figures and Tables

**Table 5:** Summary of the effectiveness of glucagon-like peptide-1 receptor agonist (GLP-1 RA) and dipeptidyl peptidase-4 inhibitor (DPP-4i) administration in cardiovascular disease prevention and/or treatment.

	DPP-4is	GLP-1 Ras
MACE	No significant MACE reduction; *possible beneficial effects in patients younger than 65 years*	**Proven MACE reduction**
Atherosclerosis and atherothrombosis	→ Decreased expression of atherosclerotic plaque markers; → improved endothelial function **= lower cardiovascular risk.** *However, fewer studies with proven molecular effects.*	→ Decreased expression of atherosclerotic plaque markers; → improved endothelial function; → enhanced mitochondrial function **= lower cardiovascular risk.**
Cardiac arrhythmias	→ Reduced fibrosis in animal studies. No difference in the risk of new-onset AF between DPP-4is and GLP-1 RAs. *Important: might be associated with the highest proportion of AF events in diabetic patients.*	→ Reduced fibrosis in animal studies. No difference in the risk of new-onset AF between DPP-4is and GLP-1 RAs. *Could potentially be an alternative or adjunctive treatment for cardiac fibrosis-related conditions*.
Myocardial infarction and heart failure	→ No reduction in natriuretic peptides; → Enhance remodeling of the extracellular matrix. ***Important:*** *elevated (saxagliptin) or no elevated risk of HF-related hospitalizations in clinical studies.*	→ Reduction in natriuretic peptides; → Enhancement of extracellular matrix remodeling; Improvement of the quality of life in HF patients. **Lower risk of hospitalization due to cardiovascular disease in T2DM patients;** **recommended for lowering the risk of MI.**

Text in bold indicates clinically proven or recommended findings. Text in italics indicates uncertain, debated, or cautionary points.

## Data Availability

No new data were created or analyzed in this study.

## Abbreviations

ADA	American Diabetes Association
Akt	Protein kinase B
AMP	Adenosine monophosphate
AMPK	AMP-activated protein kinase
AMPKα2	AMP-activated protein kinase alpha 2
AngII	Angiotensin II
ANP	Atrial natriuretic peptide
apoA-I	Apolipoprotein AI
apoB	Apolipoprotein B
apoE	Apolipoprotein E
ASCVD	Atherosclerotic cardiovascular disease
ATP	Adenosine triphosphate
BH4	Tetrahydrobipterin
BMI	Body mass index
BNP	Brain natriuretic peptide
CAD	Coronary artery disease
cAMP	Cyclic adenosine monophosphate
CD	Cerebrovascular disease
cGMP	Cyclic guanosine monophosphate
CHF	Congestive heart failure
CMECs	Cardiac microvascular endothelial cells
COX-2	Cyclooxygenase-2
CVD	Cardiovascular disease
DBP	Diastolic blood pressure
DCD	Donation after circulatory death
DM	Diabetes mellitus
DNA	Deoxyribonucleic acid
DPP-4	Dipeptidyl peptidase-4
DPP-4i	Dipeptidyl peptidase-4 inhibitor
Drp-1	Dynamin-related protein-1
ECM	Extracellular matrix
eGFR	Estimated glomerular filtration rate
eNOS	Endothelial nitric oxide synthase
ERK1/2	Extracellular signal-regulated kinase 1/2
ET-1	Endothelin-1
Ex-4	Exendin-4
FBG	Fasting blood glucose
FoxO1	Forkhead box protein O1
GIP	Gastric inhibitory polypeptide
GLP-1 RA	Glucagon-like peptide-1 receptor agonist
GLP-1R	Glucagon-like peptide-1 receptor
GTP	Guanosine triphosphate
GPx	Glutathione peroxidase
HAECs	Human aortic endothelial cells
HbA1c	Hemoglobin A1c
HF	Heart failure
HFrEF	Heart failure with reduced ejection fraction
I/R	Ischemia/reperfusion
ICAM-1	Intercellular adhesion molecule 1
IL-1β	Interleukin-1 beta
IL-6	Interleukin 6
iNOS	Inducible nitric oxide synthase
IRS1	Insulin receptor substrate-1
IκBα	Inhibitor of kappa B alpha
JNK-1	c-Jun N-terminal kinase 1
KLF2	Krüppel-like factor 2
LDL	Low-density lipoprotein
LDL-C	Low-density lipoprotein cholesterol
LOX-1	Lectin-like oxidized low-density lipoprotein receptor 1
LPS	Lipopolysaccharide
LVEF	Left ventricular ejection fraction
lyso-PC	Lysophosphatidylcholine
MACE	Major adverse cardiovascular event
MCP-1	Monocyte chemoattractant protein 1
MI	Myocardial infarction
miR-21/PTEN/PI3K	microRNA-21/phosphatase and tensin homolog/phosphoinositide 3-kinase
MMP	Matrix metalloproteinase
MNCs	Mononuclear cells
mRNA	Messenger ribonucleic acid
NADPH	Nicotinamide adenine dinucleotide phosphate
NF-κB	Nuclear factor kappa-light-chain-enhancer of activated B cells
NO	Nitric oxide
NOS	Nitric oxide synthase
NOX4	Nicotinamide adenine dinucleotide phosphate oxidase 4
Nrf2	Nuclear factor erythroid 2-related factor 2
Ox-LDL	Oxidized low-density lipoprotein
PAD	Peripheral artery disease
PAF-AH	Plasma platelet-activating factor acetylhydrolase
p-AMPK	Phosphorylated AMP-activated protein kinase
PC	Phosphatidylcholine
PCOS	Polycystic ovary syndrome
p-eNOS	Phosphorylated endothelial nitric oxide synthase
PI3K	Phosphoinositide 3-kinase
p-IκBα	Phosphorylated inhibitor of kappa B alpha
PKA	Protein kinase A
PKG	Protein kinase G
PKG/PKCε/ERK1/2	Protein kinase G/protein kinase C epsilon/extracellular signal-regulated
PMNs	Polymorphonuclear leukocytes
p-NF-κB	Phosphorylated nuclear factor kappa-light-chain-enhancer of activated B cells
pNOS3/NOS3	Phosphorylated nitric oxide synthase 3/nitric oxide synthase 3
RAGE	Receptors of advanced glycation end product
ROCK	Rho-associated coiled-coil containing protein kinase
ROCK-2	Rho-associated coiled-coil containing protein kinase 2
ROS	Reactive oxygen species
SBP	Systolic blood pressure
sGC	Soluble guanylyl cyclase
SGLT2i	Sodium–glucose cotransporter 2 inhibitor
sICAM	Soluble intercellular adhesion molecule
SMC	Smooth muscle cell
SOCS-3	suppressor of cytokine signaling 3
sVCAM	Soluble vascular cell adhesion molecule
T2DM	Type 2 diabetes mellitus
TC	Total cholesterol
TGF-β1	Transforming growth factor beta 1
TIMP	Tissue inhibitor of metalloproteinase
TLR2	Toll-like receptor 2
TLR4	Toll-like receptor 4
TNF	Tumor necrosis factor
VCAM-1	Vascular cellular adhesion molecule-1
